# The digitalization aliens

**DOI:** 10.3205/zma001348

**Published:** 2020-11-16

**Authors:** Lisa Schmitz, Jana Aulenkamp, Daniel Bechler, Jonah Grütters

**Affiliations:** 1 bvmd Bundesvertretung der Medizinstudierenden in Deutschland e.V., Berlin, Germany

**Keywords:** digitalization, medical students, interdisciplinary, digital teaching

## Abstract

Digitization in medical education opens up numerous exciting new possibilities. It is the task of those responsible for teaching to take advantage of this opportunity and use digitization as teaching content, but also as a design option for existing teaching structures. Only through up-to-date, longitudinal knowledge transfer a change be initiated and, with the help of innovative teaching and learning concepts, teachers and students can be empowered to achieve this. The aim is to evaluate, critically question and integrate digitization into the overall context of healthcare.

## Zusammenfassung

Digitalisierung in der medizinischen Ausbildung eröffnet zahlreiche spannende, neue Möglichkeiten. Dabei ist es Aufgabe der Lehrverantwortlichen diese Chance zu nutzen und die Digitalisierung als Lehrinhalt, aber auch als Gestaltungsmöglichkeit für bestehende Lehrstrukturen zu nutzen. Nur durch eine aktuelle, longitudinale Wissensvermittlung kann man einen Wandel einläuten und mithilfe von innovativen Lehr- und Lernkonzepten Dozierende und Studierende dafür befähigen. Ziel ist das Bewerten, kritische Hinterfragen und Einordnen von der Digitalisierung in den Gesamtkontext Gesundheitswesen.

## Prologue

We all dream of a future in which we live together peacefully: We dream of a happy ending from the movie we saw on Netflix last night, about aliens landing on our planet and not attacking us but wanting to make friends. Moreover, secretly we ask ourselves the question whether such a happy end is only reserved for the utopian film world. 

In the world of medicine, the arrival of the digitalization aliens is still observed with great scepticism. Visits from seemingly distant galaxies seem threatening and alien. All too often, this leads to the fact that as an Extra-terrestrial only observes the goings-on instead of reaching out his hand as a courageous astrophysicist of history himself. A world could remain hidden, which would enrich the future and the coexistence considerably [1]. 

## Act one

The arrival of an alien digitizing species was foreseen long ago by astrophysicists. When these unknown creatures one day actually landed in the world of astrophysicists, they saw this as a natural development of an ever-changing universe and eagerly explored their integration in areas such as supply, research, communication and politics. 

Over time, the alien species opened up ever new, unusual, challenging but promising possibilities for them. In situations that previously seemed hopeless and without prospects, they were able to demonstrate new networked, technically advanced solutions. 

It is now the task of astrophysicists to respond to these changes. By critically reflecting, they recognize the great opportunities of this development and are at the same time aware of their responsibility and the risks for their world. They no longer want to be silent observers, but to actively shape their world. Bridges must be built between the galaxies and spaces of encounter and exchange must be created. 

## Act two

But even if the added value of the aliens was clear to many experts, the astrophysicists did not immediately succeed in bringing the two galaxies closer together and connecting them. On the contrary: large parts of the digitized galaxy remained unknown to the local one, seeming light years away. The enormous potential, the new perspectives, possibilities, and the helpfulness of the new species were often not seen [2]. 

However, with time, the desire to get in touch with this new world sprouted in more and more people. Unfortunately, the astrophysicists had to realize that the inhabitants of their world lacked the abilities to understand the new species [3]. They began to teach digital skills that enabled them to get to know each other and engage in an intensive dialogue [4].

They convinced their fellow human beings that learning this new language was essential to ensure an equal and independent coexistence. Only those who understand their counterparts easily can interpret their statements and make their own decisions about the consequences [5].

The astrophysicists also noted that it is important to teach each generation this ability to communicate, individually adapted to their background. They placed particular emphasis on enabling the young generation to understand the new species and its language with confidence, so that they could take their future into their own hands [6], [7].

## Third act

They already taught the language of digitization in schools and universities, and were thus able to prevent exclusion and hostility even among the youngest children through broad-based basic knowledge. Through longitudinal integration, they created knowledge transfer that built on each other and a continuous debate [8]. They also gave other lecturers the opportunity to deal with the new species and to find out about the personal added value. For the galaxy of digitization was large and colourful and offered different benefits, but also dangers, in very different areas of the world. This is something that the lecturers should also be able to learn and pass on to their students. However, it was important for every lecturer to be aware that digitization is constantly changing, in addition to its diversity. It exists not only in textbooks, but also in the reality of everyday life. A large part of the skills taught therefore always consisted of critical thinking and an open attitude. 

In order to further the quest for optimal digital teaching, the astrophysicists selected outstanding projects at locations of particularly successful integration. On the one hand, they promoted these initiatives locally and, on the other hand, ensured that all other locations were also able to set up digitization projects of this kind [9]. Catalogues were published, showing methods to promote change and integration throughout the galaxy [10]. Here they listed the following elements, among others:

1. **multiplier workshops** for lecturers [11]
2. **peer and co-teaching** for students [12]
3. **innovative teaching** formats for all [13]
4. **flexible curricula** to be able to follow the rapid changes [14]
5. **interdisciplinary teaching concepts** [15]

The students developed a **sound basic understanding and basic digital skills**, which not only helped in theory, but were also of great practical importance in their everyday life and later in their jobs [16], [17]. Novel developments such as information processing systems or supply-relevant apps [18] were better understood and thus found more efficient applications in practice. Legal and ethical questions that arose for the first time in digital contexts could be better understood and answered more holistically. They understood that changes also entail risks, which made it essential to learn how to **evaluate, critically question and integrate them into the overall context**. Finally yet importantly, the understanding of new technologies helped them to apply the acquired skills even better in the real world [19], [20], [21]. The young generation was thus enabled to act independently and solution-oriented in the future and to promote a responsible integration of the alien species digitization [22]. 

## Fourth act

The brave astrophysicists who dared to see the good in the new species were rewarded for their years of effort and were able to make a decisive contribution to their dreamed happy ending. Last but not least, the teaching of digital basic skills in all generations, especially the youngest, was a decisive key to the success of the astrophysicists. 

## Epilogue

Not surprisingly, there comes a point when it can be said with a lot of energy that this happy ending is not only possible in the Netflix movie from last night. Everybody has the choice repeatedly between the role of the astrophysicist and that of the silent observer. Digitalization determines our everyday and professional life. It is an immanent and essential part of our present and future, and this fact must be conveyed already during studies and enable future physicians to make an independent choice of their role as early as possible. 

## Competing interests

The authors declare that they have no competing interests. 
